# Functional Analysis in Mouse Embryonic Stem Cells Reveals Wild-Type Activity for Three *Msh6* Variants Found in Suspected Lynch Syndrome Patients

**DOI:** 10.1371/journal.pone.0074766

**Published:** 2013-09-10

**Authors:** Eva A. L. Wielders, Hellen Houlleberghs, Gözde Isik, Hein te Riele

**Affiliations:** Division of Biological Stress Response, The Netherlands Cancer Institute, Amsterdam, The Netherlands; Ohio State University Medical Center, United States of America

## Abstract

Lynch syndrome confers an increased risk to various types of cancer, in particular early onset colorectal and endometrial cancer. Mutations in mismatch repair (MMR) genes underlie Lynch syndrome, with the majority of mutations found in *MLH1* and *MSH2*. Mutations in *MSH6* have also been found but these do not always cause a clear cancer predisposition phenotype and *MSH6*-defective tumors often do not show the standard characteristics of MMR deficiency, such as microsatellite instability. In particular, the consequences of *MSH6* missense mutations are challenging to predict, which further complicates genetic counseling. We have previously developed a method for functional characterization of *MSH2* missense mutations of unknown significance. This method is based on endogenous gene modification in mouse embryonic stem cells using oligonucleotide-directed gene targeting, followed by a series of functional assays addressing the MMR functions. Here we have adapted this method for the characterization of *MSH6* missense mutations. We recreated three *MSH6* variants found in suspected Lynch syndrome families, MSH6-P1087R, MSH6-R1095H and MSH6-L1354Q, and found all three to behave like wild type MSH6. Thus, despite suspicion for pathogenicity from clinical observations, our approach indicates these variants are not disease causing. This has important implications for counseling of mutation carriers.

## Introduction

Lynch Syndrome (LS), also called hereditary non-polyposis colorectal cancer (HNPCC), is an autosomal dominant disorder that is characterized by early onset cancer of the colorectum and endometrium. It furthermore confers an increased risk for cancers of the ovary, small intestine, stomach, ureter, renal pelvis, brain and sebaceous glands [1]. Tumors often show a high rate of microsatellite instability (MSI). The majority of LS cases is caused by inherited mutations in the DNA mismatch repair (MMR) genes *MLH1* and *MSH2* (70-80% of all LS-associated colorectal cancer (CRC) cases). Mutations in the MMR genes *MSH6* and *PMS2* account for the remaining 20-30% of LS-associated tumors [2,3]. MMR gene mutation carriers generally have an up to 10-fold increased lifetime risk of developing CRC (70-80%) and endometrial cancer (40-60%) compared to the general population [4].

In contrast to families carrying *MLH1* and *MSH2* mutations, families carrying mutations in *MSH6* often do not fulfill the criteria for LS diagnosis. Tumors in MSH6 mutation carriers frequently show no or low MSI and the observed instability is generally restricted to mononucleotide markers [5–7]. When compared to *MLH1* and *MSH2* mutation carriers, the age of onset is generally later for *MSH6* mutations carriers (approximately 10 years) and they have a lower risk for developing CRC [2,3]. There are reports of increased frequency of endometrial cancer in *MSH6* mutation carriers versus *MSH2* mutation carriers [8]; however, two large studies found no difference [2] or even a decreased [3] endometrial cancer incidence in patients carrying a mutation in *MSH6*.

The different clinical presentation of patients carrying a mutation in *MLH1* or *MSH2* and patients with *MSH6* mutations may be partially explained by the different function of these genes in the MMR pathway. MMR plays an important role in the maintenance of genomic stability via three main functions [9]: (1) recognition and repair of mis- and unpaired bases; (2) suppression of recombination between homologous but not identical DNA sequences; (3) induction of apoptosis in response to certain DNA damaging chemotherapeutics such as methylating agents and 6-thioguanine (6-TG). The mismatch repair reaction is initiated by binding of the MSH2/MSH6 (MutSα) or MSH2/MSH3 (MutSβ) heterodimer to a mismatch, a small loop of unpaired bases or to one of several DNA adducts. An MLH1/PMS2 or MLH1/MLH3 heterodimer is then recruited to enable further repair. MSH2/MSH6 mainly recognizes single mis- or unpaired bases whereas MSH2/MSH3 has a preference for loops of multiple unpaired bases. This may explain the low rate of MSI on dinucleotide markers in MSH6-deficient tumors. Also the lower CRC risk and later age of onset in *MSH6* versus *MLH1* and *MSH2* mutation carriers may be due to the partial redundancy of MutSα and MutSβ [10].

The majority of MMR gene mutations found in LS families are inactivating mutations such as frameshifts, nonsense mutations and deletions. In addition, missense mutations are frequently detected and their number is increasing. However, the implications of such variants are much more difficult to interpret. To date, the number of *MSH6* missense mutations listed in the Leiden Open Variation Database (LOVD) lies around 170 [11]. Co-segregation analysis is often difficult for these variants due to small family size and, in particular in the case of *MSH6*, incomplete penetrance. This complicates reliable classification and hence, these so-called variants of uncertain significance (VUS) pose a problem to the clinic.

Current classification methods include computational analyses that estimate the severity of an amino acid substitution from evolutionary conservation and physicochemical differences [12,13]. It is however difficult to deduce reliable classifications from these algorithms and they do not always allow assessment of MSH6 variants [12]. Also *in vitro* assays are utilized to study the effect of missense mutations on the MMR pathway. In the majority of such studies, the wild-type and mutant protein are ectopically expressed and compared for biochemical properties such as ATPase activity, mismatch binding capacity and protein interactions. The repair activity is mostly studied using *in vitro* repair assays or by measuring the capacity to rescue a MMR-deficient strain or cell line [14]. A limitation of these assays is the use of overexpression, which can mask a partial defect, and the frequent use of distantly related species such as yeast and *Escherichia coli*, whose MMR genes share only partial homology with the corresponding human sequences. Furthermore, these assays mainly look at the mutator phenotype and often do not examine the prevention of homologous recombination and the induction of the DNA damage response.

We have recently described a method for the functional characterization of *MSH2* VUS that overcomes these limitations. Briefly, the gene variant is recreated at the endogenous locus of mouse embryonic stem cells (ESC) by oligonucleotide-directed gene modification [15], which is followed by analysis of the three main MMR functions using a set of functional assays [16]. Here we show that this method can be adapted to study the functional consequences of *MSH6* VUS that have been found in suspected LS patients.

## Materials and Methods

### Ethics statement

The genetic modification of *Msh6* in murine ESCs fell under the approval of the Dutch government (ministry of infrastructure and the environment) by a permit assigned to the Netherlands Cancer Institute.

### Embryonic stem cell lines

Wild-type mouse ESCs were derived from the 129Ola-derived cell line E14, originally provided by M. Hooper [17]. *Msh2* ^*-/-*^, *Msh6* ^*-/-*^ ESCs have been described in ref [10,18] and [10], respectively.

### Generation of codon substitutions in Msh6

The *Msh6* mutant ESC lines were generated by oligonucleotide-directed gene modification (‘oligo targeting’), essentially as described [15]. Briefly, we transfected ESCs with a single-stranded DNA oligonucleotide (see Figure S1) after establishing transient down regulation of MSH2 or MLH1. Transfected cells were expanded and plated at 5000 cells/well on 96-well plates. Mutation-specific PCR was used to identify pools containing mutated cells as described [16]. A positive well was subcloned in subsequent rounds by limiting dilution in pools of 1000, 100 and 1 cell/well. Mutations P1085R, R1093H and L1352Q were made in *Msh6* ^*+/+*^ ESCs; mutation G1137S was made in *Msh6* ^*+/-*^ cells. Once a clonal cell line was established, cDNA was made using an oligo dT primer. We then amplified a cDNA fragment containing the modified codon by PCR. The resulting PCR product was cloned into the pGEM^®^-T Easy vector (Promega) and sequenced to verify the presence of the mutation using vector primers T7 and SP6. Primer sequences are available upon request. For mutant G1137S, sequencing was directly performed on the PCR product.

The generation of *de novo* cell lines was performed under Dutch legislation.

### Duplication of the targeted allele

For duplication of the *Msh6* ^*PR*^, *Msh6* ^*RH*^ and *Msh6* ^*LQ*^ alleles, we used the *Pim1* targeting method as described [16]. Briefly, we targeted the heterozygous mutant cell lines with a *Pim1-neo* targeting construct [19] to mark chromosome 17, carrying the wild-type or mutated *Msh6* sequence, by a *neo* gene and subsequently subjected several clones of each cell line to high G418 concentrations. Picked colonies were screened for duplication of the neo-marked chromosome (and concomitant loss of the non-marked chromosome) using a PCR specific for the targeted *Pim1* locus.

We subsequently screened resulting *Pim1* ^*neo/neo*^ colonies for duplication of the mutated *Msh6* allele via sequencing. We amplified the genomic DNA of the wild-type, heterozygous and homozygous mutant cell lines and performed a sequencing reaction on the purified PCR products. Primer sequences are available upon request.

### Western blot analysis

Cells were lysed in a buffer containing 150 mM NaCl, 50 mM Hepes pH 7.5, 5 mM EDTA, 0.1% NP-40, 5 mM NaF, 0.5 mM vanadate, 20 mM β-glycerolphosphate and 1 tablet complete protease inhibitor cocktail (Roche) per 50 ml. Protein extracts from 1.5 x 10^5^ ESCs were separated by 3-8% Tris-Acetate gels (NuPAGE^®^) using the NuPAGE^®^ electrophoresis system and transferred to nitrocellulose membrane. We used rabbit polyclonal antibodies as primary antibodies to detect MSH6 [10] (1:500) and MSH2 [20] (1:500), and a mouse monoclonal antibody to detect γ-Tubilin (GTU-88, Sigma-Aldrich). Peroxidase-conjugated goat anti-rabbit IgG and goat anti-mouse IgG (BioSource International) were used as a secondary antibody. Signals were visualized with enhanced chemiluminescence and quantified using an Epson V750Pro scanner.

### Generation of Msh6^mut/-^ heterozygous cell lines

Cells of the three homozygous mutant cell lines *Msh6* ^*PR/PR*^ *, Msh6* ^*RH/RH*^ *, Msh6* ^*LQ/LQ*^ were targeted with a conventional *Msh6* targeting vector [10]. The loss of one of the *Msh6* alleles was verified by Southern blot analysis [10].

### Hprt mutation assay

Single cells of the homozygous mutant cell lines *Msh6* ^*PR/PR*^ *, Msh6* ^*RH/RH*^ *, Msh6* ^*LQ/LQ*^, the heterozygous mutant cell lines *Msh6* ^*PR/-*^ *, Msh6* ^*RH/-*^, *Msh6* ^*LQ/-*^, *Msh6* ^*GS/-*^ and the control cell lines Msh2^+/+^, *Msh2* ^*-/-*^, *Msh6* ^*-/-*^ and *Msh6* ^*+/-*^ were expanded to 10^9^ cells. Each expanded clone was plated onto three 150 mm gelatin-coated tissue culture plates at 1.5 x 10^6^ cells/plate. The next day, 6-TG was added at a final concentration of 10 μg/ml. After 10 days, the resistant colonies were counted.

### Microsatellite instability assay

Three single-cell clones of each mutant and control cell line were expanded to 10^9^ cells. Of each of the expanded cultures we generated 32 subclones and isolated genomic DNA. The length of two or three different dinucleotide microsatellite markers (D18Mit19, D7Mit17, D14Mit15) was analyzed by PCR analysis in case of the homozygous mutant cell lines and the control cell lines Msh2^+/+^, *Msh2* ^*-/-*^ and *Msh6* ^*-/-*^. Microsatellite instability in heterozygous *Msh6* ^*mut/-*^ and *Msh6* ^*+/-*^ ESC lines was measured using two mononucleotide markers (mBAT-26 and mBAT37) [21].

### Homologous recombination assay

To measure mismatch-directed anti-recombination activity of MMR, we compared the targeting efficiency of two constructs, 129Rb-pur (100% homologous) and Balb/cRb-pur (99.4% homologous) [22]. The targeting and subsequent analysis was performed in the *Msh6* ^*PR/PR*^ *, Msh6* ^*RH/RH*^ and *Msh6* ^*LQ/LQ*^ cell lines as described [23].

### Sensitivity to MNNG and 6-TG

The homozygous mutant cell lines, *Msh6* ^*PR/PR*^ *, Msh6* ^*RH/RH*^, *Msh6* ^*LQ/LQ*^, the heterozygous cell lines *Msh6* ^*PR/-*^ *, Msh6* ^*RH/-*^, *Msh6* ^*LQ/-*^, *Msh6* ^*GS/-*^ and the control cell lines, Msh2^+/+^, *Msh2* ^*-/-*^, *Msh6* ^*-/-*^ and *Msh6* ^*+/-*^ were plated onto irradiated mouse embryonic fibroblast feeder layers at a density of 500 cells/1.8 cm^2^. The next day we treated the cells for 1 hour with 0-40 μM MNNG or 6-TG and after 4 days we counted the number of surviving colonies.

In the case of MNNG exposure, cells were cultured in the presence of 40 μM *O* ^6^-benzylguanine, starting from 1 hour prior to MNNG exposure until counting of the colonies. *O* ^6^-benzylguanine inhibits the removal of methyl groups from the *O* ^6^ position of guanine by endogenous *O* ^6^-methylguanine-methyltransferase activity.

## Results

### Selection of MSH6 variants of uncertain significance

We have selected three *MSH6* VUS that have been found in suspected Lynch syndrome families: hMSH6-P1087R, hMSH6-R1095H and hMSH6-L1354Q. The hMSH6-P1087R substitution was found in a family that did not fulfill the criteria for hereditary colon cancer but did show familial clustering of LS-associated tumor types suggesting the presence of a pathogenic mutation [8]. In other families, the 1087 proline residue was found mutated to serine [24,25] or threonine [26], which could indicate functional importance of this proline residue. The hMSH6-R1095H and hMSH6-L1354Q missense mutations were identified in two separate families, both suspected of LS but not fulfilling the criteria. In both families, the index patients also carried the same *MSH2* missense mutation, hMSH2-I145M, but a previous functional study was unable to identify the disease causing mutation in these families [27]. Since it is unknown which mutation was retained in the tumor, we decided to recreate the *MSH6* variants in cells expressing wild-type *MSH2* in order to determine their activity in MMR.

### Generation of Msh6^mut/mut^ mouse ESCs

**Figure 1:**
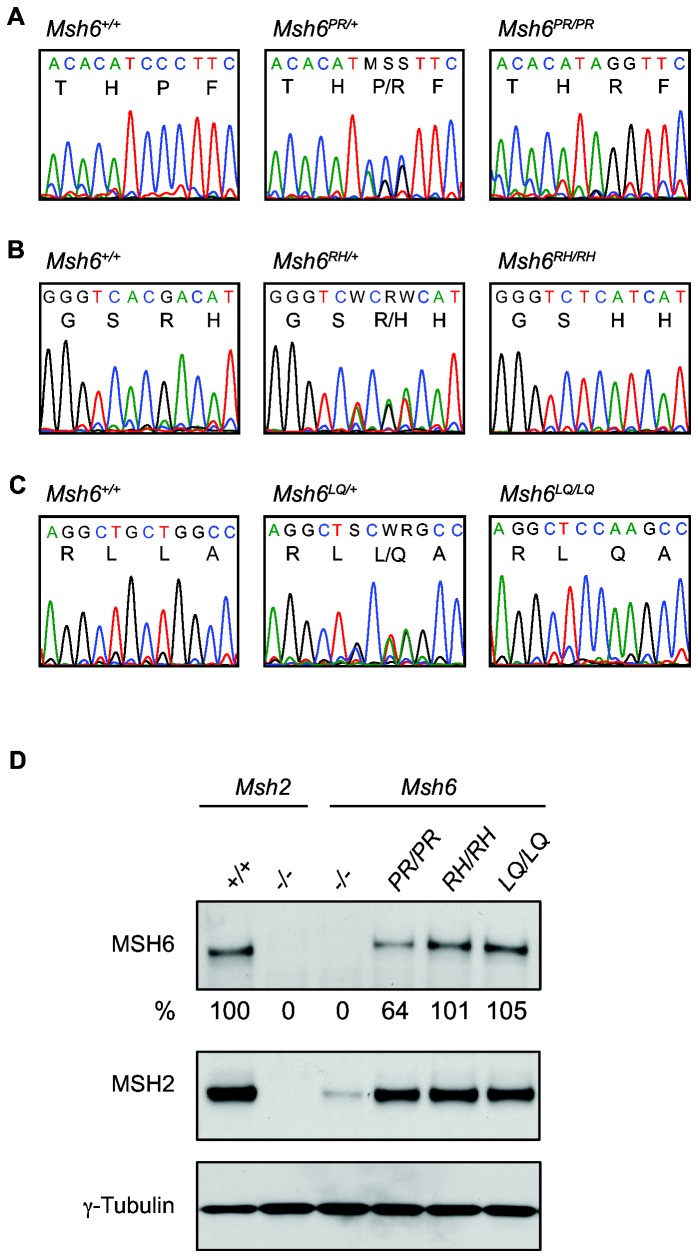
Generation of homozygous *Msh6* mutant ESC lines. Sequence analysis of (**A**) *Msh6* ^*+/+*^, *Msh6* ^*PR/+*^ and *Msh6* ^*PR/PR*^ genomic DNA, (**B**) *Msh6* ^*+/+*^, *Msh6* ^*RH/+*^ and *Msh6* ^*RH/RH*^ genomic DNA and (**C**) *Msh6* ^*+/+*^, *Msh6* ^*LQ/+*^ and *Msh6* ^*LQ/LQ*^ genomic DNA. Single letter amino acid codes are given below the sequence. (**D**) Whole cell lysates were analyzed for MSH6 and MSH2. γ-Tubulin was used as a loading control. ‘-‘ indicates a knockout allele. The relative percentages of MSH6 levels are indicated.

We used oligo targeting [15] to introduce the equivalents of the three VUS into the genome of mouse ESCs, resulting in *Msh6* ^*P1085R/+*^ *, Msh6* ^*R1093H/+*^ and *Msh6* ^*L1352Q/+*^ ESC lines (note the two position difference between the human and mouse codon numbering). The oligonucleotides used for gene modification are shown in Figure S1. We further refer to these cells lines as *Msh6* ^*PR/+*^ *, Msh6* ^*RH/+*^ and *Msh6* ^*LQ/+*^, respectively. In order to study the effect of the mutations on MMR functions, the wild type allele had to be inactivated. Using a *Pim1* targeting vector [23] we introduced *neo* into the *Pim1* gene, which is located centromerically of *Msh6* on chromosome 17 [16]. Subsequently, the neo-labeled chromosome was duplicated at the expense of the non-labeled chromosome by selecting cells at high G418 concentrations as described in Mortensen et al. [28]. Concomitant duplication of the mutant allele and loss of the wild type *Msh6* allele was confirmed by sequencing (Figure 1A–C).

We then determined the levels of MSH6 and MSH2 in homozygous mutant ESCs (Figure 1D). The level of MSH6 in *Msh6* ^*PR/PR*^ cells appeared to be twofold lower than in *Msh6* ^*RH/RH*^ and *Msh6* ^*LQ/LQ*^ cells that both showed wild-type levels. However, in all three homozygous mutant cell lines, including *Msh6* ^*PR/PR*^, MSH2 levels were virtually normal in contrast to *Msh6*-deficient cells that showed a strongly decreased MSH2 level. This indicated normal interaction of the mutant MSH6 proteins with MSH2 as both proteins are mutually dependent for their stability [10].

### MMR capacity in Msh6^mut/mut^ cells

**Figure 2:**
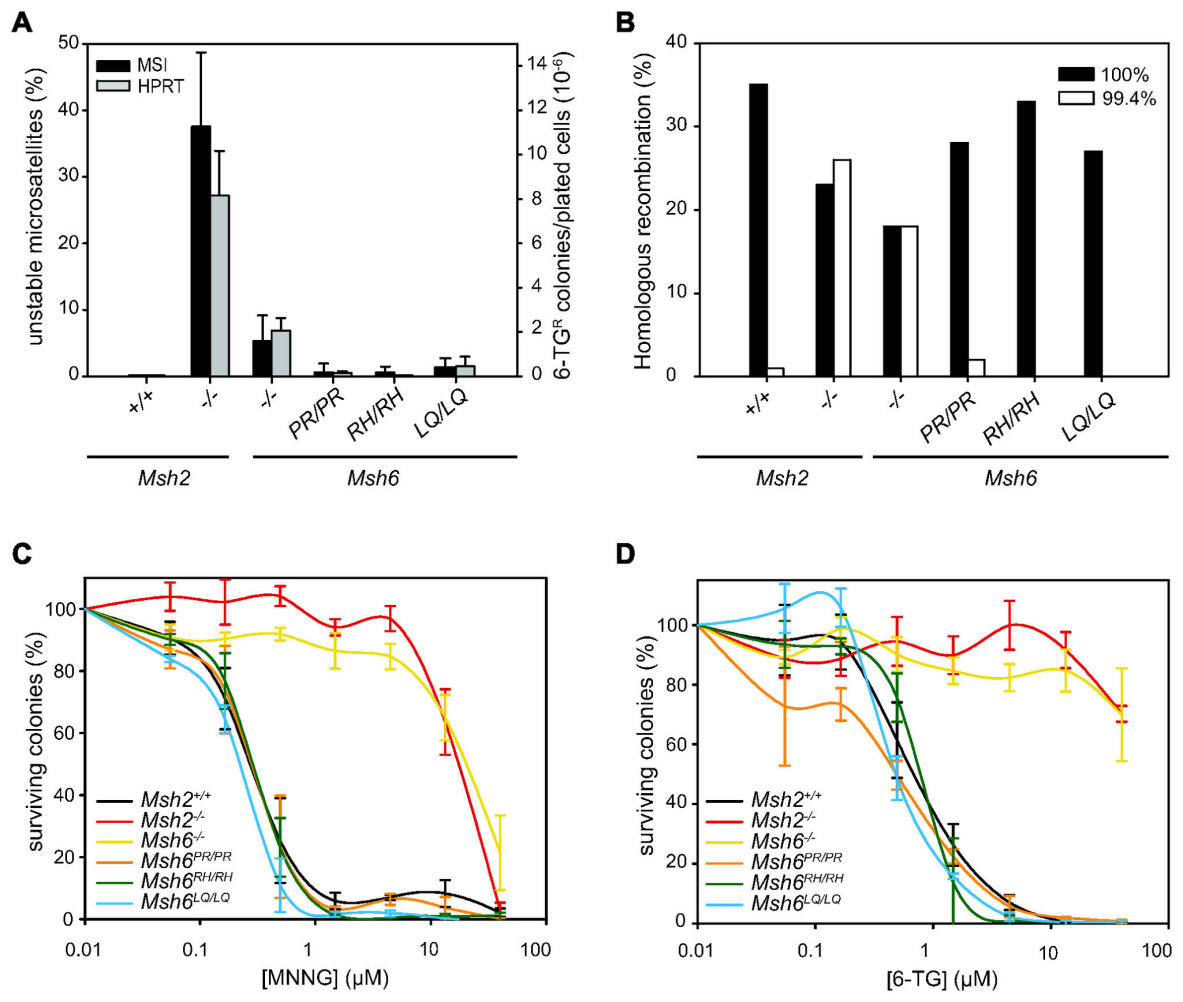
Functional analysis of *Msh6* ^*mut/mut*^ ESC lines. (**A**) Black bars show the average percentage of unstable microsatellites (left Y-axis) as measured in 96 colonies for two or three different dinucleotide markers. Error bars show standard errors, measured over two to six independent clones per cell line. The grey bars show the average number of 6-TG-resistant colonies per 10^6^ plated cells (right Y-axis). Error bars show standard errors, measured over three to six independent clones per cell line. (**B**) Targeting efficiencies are shown in mutant and control cell lines for the 100% homologous (black bars) and the 99.4% homologous (white bars) *Rb* targeting constructs. Targeting efficiencies in *Msh2* ^*+/+*^ *Msh2* ^*-/-*^ and *Msh6* ^*-/-*^ ESCs are taken from de Wind et al. [10,18] and shown as controls. (**C**) Survival of mutant and control cell lines exposed to MNNG (n=2-6). (**D**) Survival of mutant and control cell lines exposed to 6-TG (n=2-5). Error bars show standard errors from independent experiments.

To examine the effect of the introduced mutations on MMR functionality, we used three cellular assays. In order to study the mutator phenotype we looked both at slippage events at microsatellite repeats and at inactivating mutation events in the *Hprt* gene. To this aim, single cells of mutant and control cell lines were expanded to 10^9^ cells. Microsatellite instability was assessed by measuring the lengths of dinucleotide repeat markers in single-cell clones derived from the expanded cultures (Figure 2A, black bars). Mutation events in the *Hprt* gene were detected by plating cells from the expanded cultures at low density in 6-thioguanine (6-TG). Surviving colonies resulted from mutations in *Hprt* (Figure 2A, grey bars). We noted that the mutation frequencies in *Msh6*-deficient cells were lower than in *Msh2*-deficient cells. This was likely due to redundant activity of the MSH2/MSH3 dimer, which can also repair small loops that result from DNA polymerase slippage errors. In the three *Msh6* mutant cell lines, the *Hprt* mutation frequencies and the levels of dinucleotide microsatellite instability were much lower than in *Msh6*-deficient cells, virtually staying at background levels. However, it should be noted that the relatively modest mutator phenotype in *Msh6*-deficient cells reduced the sensitivity of the assay.

To avoid the confounding effect of redundant MSH3 activity, we used two assays that address MMR functions in which MSH3 does not play a role. First, a gene targeting assay was used to study the capacity of mutant MSH6 to suppress recombination between homologous but not-identical sequences. This assay compares the targeting efficiencies of *Rb* targeting vectors that either shared 100% homology or 99.4% homology with the ESC *Rb* sequence [22]. We have previously shown that suppression of homologous recombination of the non-isogenic targeting vector relies on MSH6 but not on MSH3 [10]. Indeed, *Msh6*-deficient ESCs, like *Msh2*-deficient ESCs, were fully permissive for non-isogenic gene targeting, i.e., the non-isogenic vector performed as effectively as the isogenic vector (Figure 2B). In contrast, all mutants showed no or only very limited incorporation of the non-identical vector (Figure 2B). Thus, in this assay, all three missense mutations behaved like wild-type MSH6.

Secondly, the MMR repair system is known to mediate the toxicity of certain DNA damaging agents, such as methylating agents, and also this function solely relies on MSH6 activity [10]. We exposed MSH6 mutant and control cell lines to increasing concentrations of 6-TG and MNNG in a clonogenic survival assay and observed that all three cell lines were equally sensitive to MNNG and 6-TG as wild type ESCs, whereas *Msh6*-deficient ESCs were resistant to both agents (Figure 2C and D). We therefore conclude that also this MSH6-specific MMR function was intact in all three mutant cell lines.

### The effects of reducing mutant MSH6 protein levels

**Figure 3:**
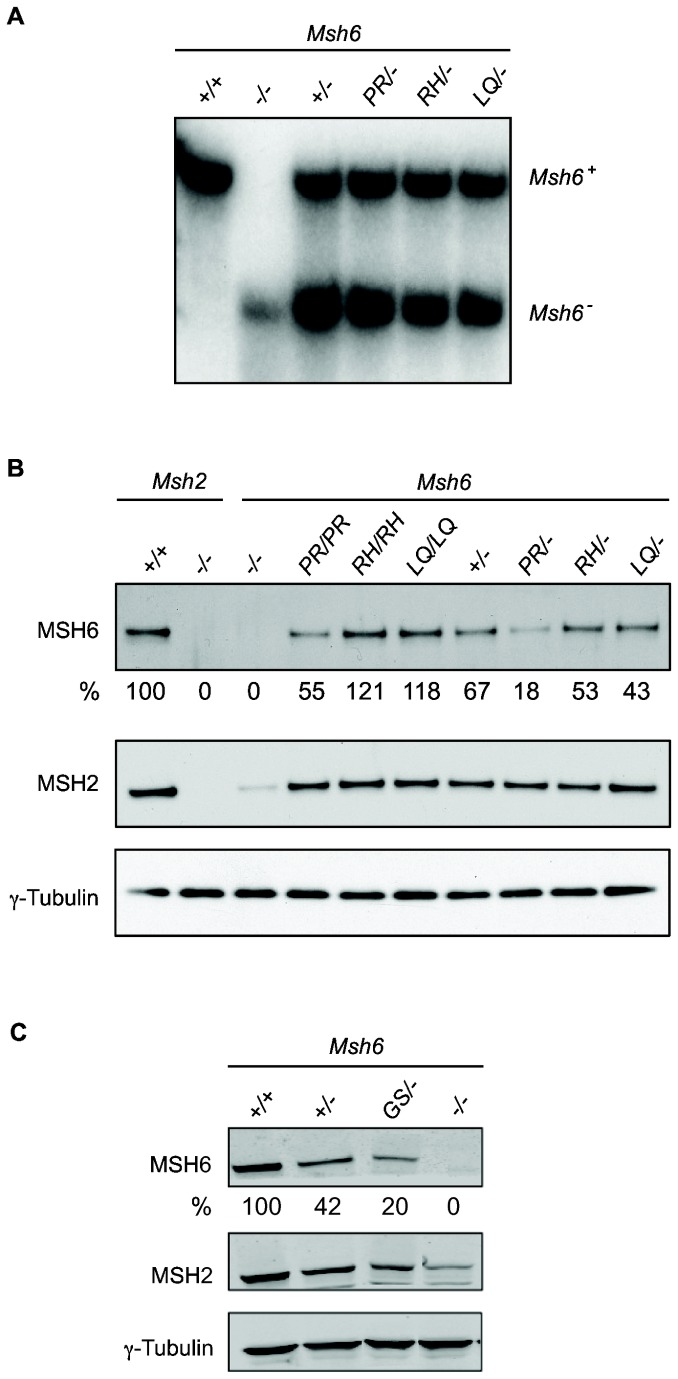
Generation of *Msh6* ^*mut/-*^ heterozygous ESC lines. (**A**) Southern blot analysis of the *Msh6* ^*mut/-*^ and control cell lines, showing loss of one of the *Msh6* alleles in *Msh6* ^*mut/-*^ cells. (**B** and **C**) Western blot analysis of mutant *Msh6* homozygous and heterozygous cell lines and controls. Whole cell lysates were analyzed for the presence of MSH6 and MSH2. γ-tubulin was used as a loading control. ‘-’ indicates a knockout allele. The relative percentages of MSH6 levels are indicated.

To exclude the possibility that the absence of a MMR defect was due to high MMR protein levels in homozygous mutant ESCs, we have also studied the MMR functionality in *Msh6* ^*mut/-*^ cells. The *Msh6* ^*mut/-*^ genotype may more closely resemble the situation in tumors in which the wild type allele is lost [29–31]. To generate *Msh6* ^*mut/-*^ ESCs, we have inactivated one of the mutant *Msh6* alleles in the homozygous *Msh6* ^*mut/mut*^ cell lines using an *Msh6* targeting vector as described before [10]. Disruption of one of the alleles was confirmed by Southern blot analysis (Figure 3A). Furthermore, to ascertain that our approach is capable of detecting a deleterious *Msh6* missense mutation, we studied an additional *Msh6* variant that was reported to be MMR defective. This variant, MSH6-G1139S, was found in a CRC patient [32] and completely failed in an *in vitro* MMR assay [33]. We targeted the wild-type *Msh6* allele in *Msh6* ^*+/-*^ ESCs to create *Msh6* ^*G1137S/-*^ ESCs as described in Figure S1. The codon substitution was confirmed by sequencing (Figure S2A).

Western blot analysis (Figure 3B) showed an approximately 2-fold reduction of MSH6 protein levels in all three *Msh6* ^*mut/-*^ cell lines compared to the respective *Msh6* ^*mut/mut*^ cell lines. In *Msh6* ^*RH/-*^ and *Msh6* ^*LQ/-*^ cells, the MSH6 protein levels were similar to those in *Msh6* ^*+/-*^ cells. The MSH6 level in *Msh6* ^*PR/-*^ cells was two to threefold lower than in the other heterozygous cell lines, which was in accordance with the reduced levels observed in *Msh6* ^*PR/PR*^ (Figure 1D). The MSH6 level in *Msh6* ^*GS/-*^ cells was reduced threefold with respect to *Msh6* ^*+/-*^ cells (Figure 3C). Since the MSH2 level in *Msh6* ^*GS/-*^ cells was higher than in *Msh6* ^*-/-*^ cells, MSH6-G1137S protein has at least retained some capacity to bind and stabilize MSH2.

**Figure 4:**
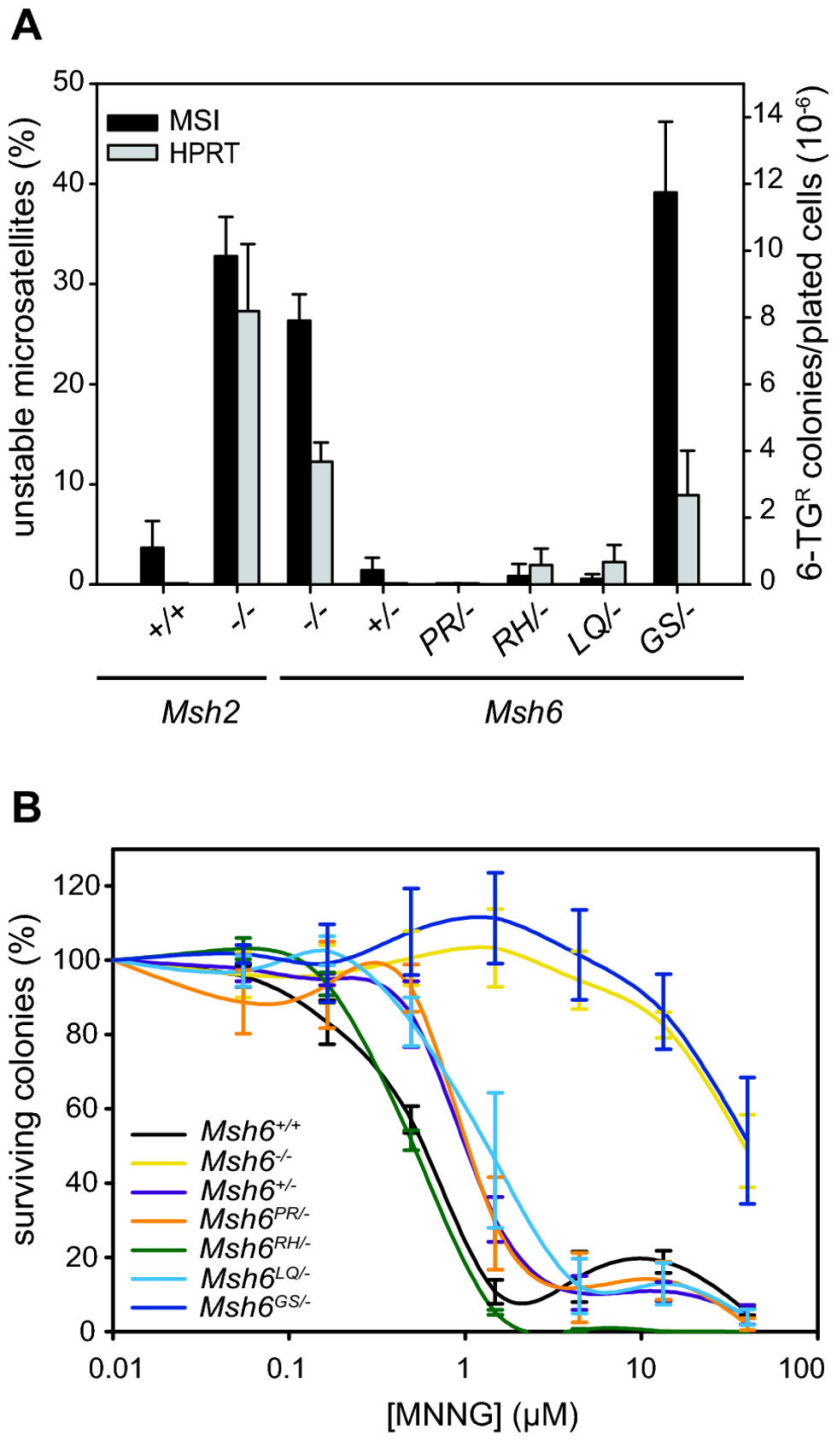
Functional analysis of *Msh6* ^*mut/-*^ heterozygous ESC lines. (**A**) Black bars show the average percentage of unstable microsatellites (left Y-axis) as measured in 96 colonies for two different mononucleotide markers. Error bars show standard errors, measured over three independent clones per cell line. Grey bars show the average number of 6-TG resistant colonies per 10^6^ plated cells (right Y-axis). Error bars show standard errors, measured over three independent clones per cell line. (**B**) Survival of mutant and control cell lines exposed to MNNG. Error bars show standard errors from three independent experiments.

To study the mutator phenotype, we performed microsatellite instability and *Hprt* mutation assays as described above. However, we now measured the length of two mononucleotide repeats instead of dinucleotide repeats. This should enhance the sensitivity of the MSI assay as readout for MSH6 activity since mononucleotide slippage events are only poorly recognized by redundant MSH2/MSH3 activity in contrast to dinucleotide slippage events [34] (see also Figure 2A). Indeed, the level of MSI at mononucleotide repeats was similar in *Msh2*- and *Msh6*-deficient ESCs (Figure 4A). Neither assay showed a mutator phenotype in *Msh6* ^*PR/-*^ cells. *Msh6* ^*RH/-*^ and *Msh6* ^*LQ/-*^ cells were also microsatellite stable; in the *Hprt* assay, only a few 6-TG-resistant colonies appeared (Figure 4A). Together, these results show effective suppression of spontaneous mutagenesis in each of these heterozygous mutant ESC lines. In contrast, in *Msh6* ^*GS/-*^ cells, MSI and mutagenesis at *Hprt* were as high as in *Msh6* ^*-/-*^ cells.

To examine the effect on the DNA damage response, we exposed the cells to MNNG. As shown in Figure 4B, *Msh6* ^*+/-*^ cells were less sensitive to this agent than wild-type cells, indicating that the MSH6 level in heterozygous cells was just below the threshold level for full sensitivity. We therefore anticipated that a minor defect in MSH6 activity in *Msh6* ^*mut/-*^ ESCs would translate into increased survival. However, we did not observe increased resistance of *Msh6* ^*PR/-*^, *Msh6* ^*RH/-*^ and *Msh6* ^*LQ/-*^ cells compared to *Msh6* ^*+/-*^ cells, indicating that each of the three MSH6 variants were as effective as wild-type MSH6 in the processing of MNNG-induced DNA damage. In contrast, *Msh6* ^*GS/-*^ cells were as resistant as *Msh6* ^*-/-*^ cells. Furthermore, also 6TG sensitivity was abrogated in *Msh6* ^*GS/-*^ cells (Figure S2B).

## Discussion

Here we show that our previously described method for the functional characterization of *MSH2* VUS [16] can be adapted to classify missense mutations found in the *MSH6* gene of suspected LS patients. Besides *Msh6* ^*mut/mut*^ ESCs, we have also generated and studied *Msh6* ^*mut/-*^ ESCs as either genotype may occur in human tumors. Both genotypes gave the same results. Furthermore, we demonstrated MSI analysis of mononucleotide repeats rather than dinucleotide repeats to better discriminate between MMR activity in *MSH6* wild-type and knockout cells. In contrast to the proven deleterious variant MSH6-G1137S, the mouse equivalents of hMSH6-P1087R, hMSH6-R1095H and hMSH6-L1354Q, behaved like wild-type MSH6 protein. Although clinical data suggested the presence of a pathogenic mutation in the respective families, our results indicate that none of the three MSH6 variants can be held responsible for cancer predisposition.

In case of the first variant, hMSH6-P1087R, we did not detect a defect in any of our assays in neither the *Msh6* ^*PR/PR*^ nor the *Msh6* ^*PR/-*^ ESC line, despite an approximately five-fold reduced mMSH6-PR protein level compared to the mMSH6 level in wild type ESCs. Our assays therefore classify this mutation as neutral. The hMSH6-P1087R variant was found in a patient who presented with a colon tumor at age 37 and had an unspecified relative with unknown mutation status who developed colon cancer at the age of 31. No MSI data was reported and the mutation was not detected in random individuals [8]. Besides the proline to arginine substitution, two other substitutions, proline to threonine and proline to serine, have been described at this position. The P1087T substitution was found in a patient with CRC at the age of 57 but not in 189 healthy controls [26]. No MSI analysis was conducted on tumor material from the index patient. *In silico* analysis using the SIFT algorithm classified both the P1087R and the P1087T substitutions as pathogenic [35]. Kariola et al. [36] studied the effects of the P1087R and the P1087T substitutions using baculovirus-expressed hMSH6 protein. Immunoprecipitation experiments showed normal binding of hMSH2 to both hMSH6-P1087R and hMSH6-P1087T; however, the level of hMSH6-P1087R protein (but not of hMSH6-P1087T) was slightly reduced. The latter is in accordance with the reduced mMSH6-PR protein we observed and is possibly due to somewhat decreased stability resulting from the presence of the charged arginine residue in a hydrophobic domain. The *in vitro* repair assay conducted in that study showed wild-type repair capacity of both hMSH6-P1087 variants, which is in agreement with the absence of a mutator phenotype in our *Hprt* and MSI assays. The proline to serine substitution has been found in a patient with CRC at the age of 39, who also had a monoallelic missense mutation in MUTYH [24]. MUTYH mutations are the cause of MUTYH-associated Polyposis (MAP) and monoallelic MUTYH missense mutations have been suggested to confer an increased CRC risk when combined with *MSH6* mutations [37]. The tumor status was MSI-low and MLH1, MSH2 and MSH6 were detected using immunohistochemistry (IHC) [24]. In summary, although the clinical data was suggestive for pathogenicity, our results, corroborating the other functional data mentioned above, indicate that the hMSH6-P1087R mutation is not disease causing.

The hMSH6-R1095H and hMSH6-L1354Q missense mutations were found in two individuals, respectively, from two separate families, both suspected of LS but not fulfilling the criteria. Interestingly, both probands also carried the same MSH2-I145M missense mutation. The MSH6-R1095H mutation carrier was member of a family in which four out of seven siblings developed CRC at the ages of 65 (proband; also CRC at age 74), 60, 60 and 76. However, the mutation status of the siblings is unknown. IHC detected MSH2, MSH6 and MLH1 expression in the tumor of the index patient. MSI was detected at two dinucleotide repeats but two other dinucleotide repeats and one mononucleotide repeat were stable and the result of a second mononucleotide repeat was unclear. The proband carrying the MSH6-L1354Q and MSH2-I145M mutations presented with CRC at the age of 53 and had a sister who developed CRC at the age of 63. Two other siblings were healthy but again, their mutation status is unknown. The tumor in the proband showed MSI at all four markers studied (two di- and two mononucleotide repeats) and IHC failed to detect both MSH2 and MSH6 expression whereas MLH1 expression was still present [27]. The finding that in both families all cancer patients were from the same generation led the authors to suggest that the mutations were inherited from both parents and that only when both were present, they increased the cancer susceptibility [27]. However, it is difficult to see how this compound heterozygosity would cause tumor predisposition as the MSH2-I145M/MSH6-L1354Q complex would only be a minority and would even be absent in tumors in case of loss of heterozygosity. Also, although the L1354 residue is located in an MSH2-MSH6 interaction region [38], the MSH2-I145M residue is not. Moreover, all combinations of baculovirus-produced wild-type and mutant human MSH2 and MSH6 performed equally well in an *in vitro* mismatch repair assay and SIFT analysis classified all three variants as neutral [35], indicating that none of the three mutations caused tumor predisposition. Our data on the MSH6 mutations supports this conclusion since we observed that the endogenously expressed mutant MSH6 proteins both behaved like wild type in our functional assays. To address the possibility that the tumor predisposition was due to the MSH2-I145M mutation, we are recreating this variant in ESCs (although it should be noted that the isoleucine residue is not conserved in mouse). It is also possible that the MSI and absence of MSH2/MSH6 staining in the tumor with the L1354Q mutation was due to a defect outside the sequenced coding regions, such as the recently identified *TACSTD1* deletion upstream of *MSH2* [39].

We have previously shown that recreating MSH2 variants at the endogenous locus of murine ESCs and subsequent cellular analysis of MMR functions is a valuable tool to study MSH2 VUS that have been found in suspected familial cancer cases. We have shown here that this method can be adapted for the study of MSH6 VUS. A fundamental question now is whether in the absence of sufficient clinical data, our approach can reliably classify MMR gene VUSs to provide guidance to clinical geneticists in the counseling of mutation carriers. A possible caveat of our approach is that mutations are studied in the context of the embryonic stem cell transcriptome, which supports high levels of MMR gene expression. Thus, the phenotype of a specific variant may be masked by an excessively high expression level. However, we consider this unlikely as a twofold reduction of wild-type MSH6 protein level in *Msh6* ^*+/-*^ cells already reduced the sensitivity of cells to MNNG (Figure 4B). Nevertheless, to study whether certain variants only exert an effect at expression levels found in tissues, we are currently introducing our MMR gene mutations in mice, which will be monitored for susceptibility to tumorigenesis.

## Supporting Information

Figure S1 **Msh6 targeting oligonucleotides.** Msh6 targeting oligonucleotides (upper case) hybridized to their complementary genomic sequence (lower case); mismatching bases in the oligonucleotides are shown in red. The codon alteration is underlined. P1085R was effectuated by substituting CCC for AGG; R1093H by ACGA for TCAC; L1352Q by GCTG for CCAA; G1137S by GGC for AGC.
(PDF)

Figure S2 **Verification and analysis of *Msh6*^*G1137S/-*^ ESCs.** (**A**) Sequence analysis of genomic DNA of *Msh6* ^*G1137S/-*^ ESCs showing the G to A substitution. Single letter amino acid codes are given below the sequence. (**B**) *Msh6* ^*G1137S/-*^ ESCs are resistant to 6TG.
(PDF)
