# Supplementary figures and images for: Functional Analysis in Mouse Embryonic Stem Cells Reveals Wild-Type Activity for Three Msh6 Variants Found in Suspected Lynch Syndrome Patients
Source: PLoS One. 2013 Sep 10;8(9):e74766. doi: 10.1371/journal.pone.0074766 (PMC3769292; doi:10.1371/journal.pone.0074766)

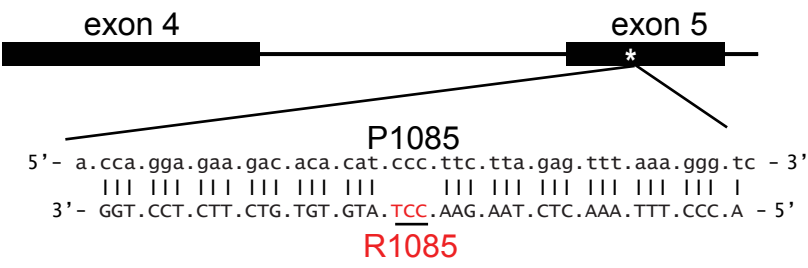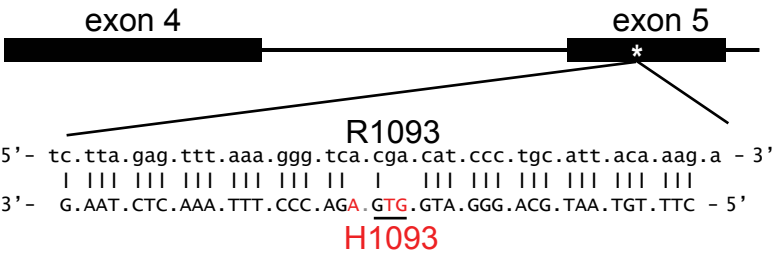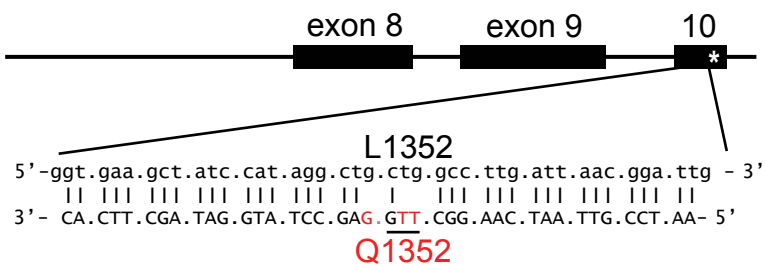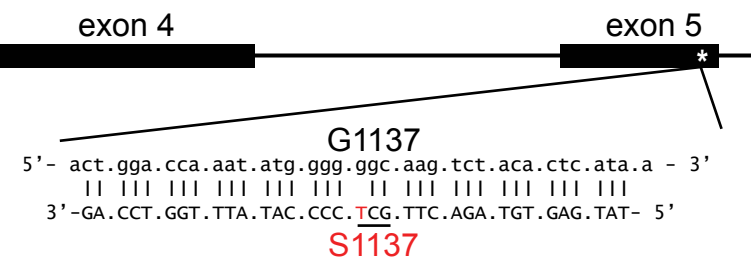

Supplement: Figure S1 — Msh6 targeting oligonucleotides. Msh6 targeting oligonucleotides (upper case) hybridized to their complementary genomic sequence (lower case); mismatching bases in the oligonucleotides are shown in red. The codon alteration is underlined. P1085R was effectuated by substituting CCC for AGG; R1093H by ACGA for TCAC; L1352Q by GCTG for CCAA; G1137S by GGC for AGC. (PDF) [file pone.0074766.s001.pdf]

A

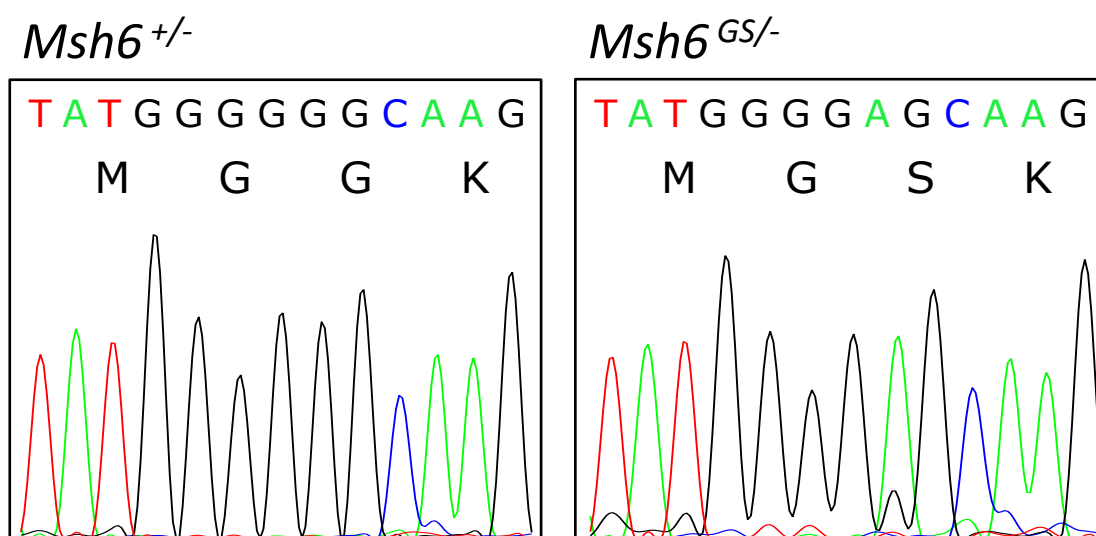

B

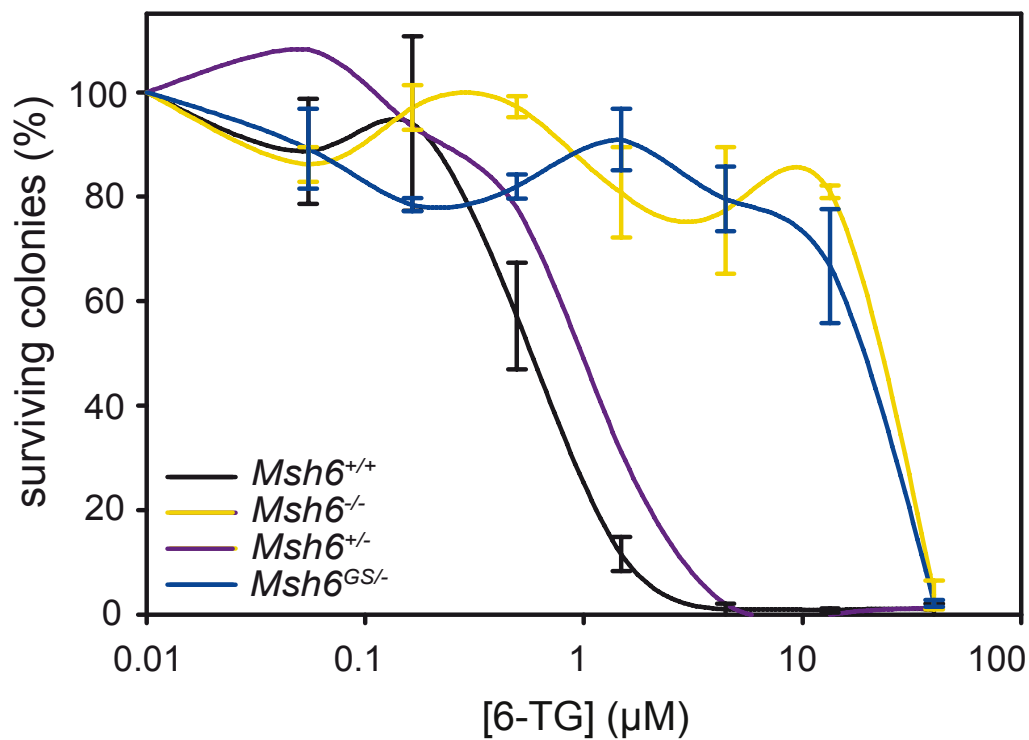

Supplement: Figure S2 — Verification and analysis of Msh6G1137S/- ESCs. (A) Sequence analysis of genomic DNA of Msh6 G1137S/- ESCs showing the G to A substitution. Single letter amino acid codes are given below the sequence. (B) Msh6 G1137S/- ESCs are resistant to 6TG. (PDF) [file pone.0074766.s002.pdf]
